# Supplementary material for: Perspectives and experiences of Covid-19: Two Irish studies of families in disadvantaged communities
Source: PLoS One. 2022 Jul 18;17(7):e0270472. doi: 10.1371/journal.pone.0270472 (PMC9292082; doi:10.1371/journal.pone.0270472)
Supplement: Attachment — Submitted filename: Response to Reviewers.pdf [file pone.0270472.s001.pdf]

Rebuttal letter

Manuscript ID: PONE-D-21-40789

Manuscript title: Perspectives and experiences of Covid-19: Two Irish studies of families in disadvantaged communities

To: PLOS ONE

Re: Response to Editor and Reviewer

Dear Editor,

We thank the Academic Editor and the Reviewer for the constructive comments on our manuscript and for the opportunity to revise it. We have carefully considered these comments and adjusted the manuscript accordingly. Please, find below our response to each point raised by the Academic Editor and Reviewer. We hope that we satisfyingly addressed them.

Sincerely,

On behalf of all authors,

Catarina Leitão

27 May 2022

Responses to Academic Editor's comments on Journal Requirements

1. Please ensure that your manuscript meets PLOS ONE's style requirements, including those for file naming. The PLOS ONE style templates can be found at https://journals.plos.org/plosone/s/file?id=wjVg/PLOSOne_formatting_sample_main_body.pdf and https://journals.plos.org/plosone/s/file?id=ba62/PLOSOne_formatting_sample_title_authors_affiliations.pdf

Response: We have checked the manuscript and filenames to ensure that they comply with PLOS ONE's style and naming requirements. We removed the italics style from the text presented in the Instruments sections. We added visible margins to all cells of the tables. Moreover, an additional private email address of the corresponding author CL on the title page has been added to ensure continuity in communication in cases of future inquiries about the manuscript. The reason is that the given institutional address will be deactivated in the near future.

We also revised the grammar of some sentences to improve clarity, and the use of capital letters and hyphenated words to ensure consistency throughout the manuscript.

2. Thank you for stating the following in the Competing Interests section:
The authors have read the journal's policy and have the following competing interests: Study 1 was conducted within a project that has received funding from the European Union's Horizon 2020 research and innovation programme under the Marie Skłodowska-Curie grant agreement No 890925. Study 2 was funded by Tusla under the Area Based Childhood funding and the Child and Youth Participation Initiatives grant.
Please confirm that this does not alter your adherence to all PLOS ONE policies on sharing data and materials, by including the following statement: "This does not alter our adherence to PLOS ONE policies on sharing data and materials." (as detailed online in our guide for authors http://journals.plos.org/plosone/s/competing-interests). If there are restrictions on sharing of data and/or materials, please state these. Please note that we cannot proceed with consideration of your article until this information has been declared.
Please include your updated Competing Interests statement in your cover letter; we will change the online submission form on your behalf.

Response: We updated the Competing Interests statement in our cover letter to confirm the adherence to all PLOS ONE policies on sharing data and materials. The complete Competing Interests statement is now the following:
Study 1 was conducted within a project that has received funding from the European Union's Horizon 2020 research and innovation programme under the Marie Skłodowska-Curie grant agreement No 890925. Study 2 was funded by Tusla under the Area Based Childhood funding and the Child and Youth Participation Initiatives grant. This does not alter our adherence to PLOS ONE policies on sharing data and materials.

3. We note that you have stated that you will provide repository information for your data at acceptance. Should your manuscript be accepted for publication, we will hold it until you provide the relevant accession numbers or DOIs necessary to access your data. If you wish to make changes to your Data Availability statement, please describe these changes in your cover letter and we will update your Data Availability statement to reflect the information you provide.

Response: We will provide the relevant accession numbers or DOIs to access the data after acceptance, before publication. We do not wish to make changes to our Data Availability statement.

4. Please review your reference list to ensure that it is complete and correct. If you have cited papers that have been retracted, please include the rationale for doing so in the manuscript text, or remove these references and replace them with relevant current references. Any changes to the reference list should be mentioned in the rebuttal letter that accompanies your revised manuscript. If you need to cite a retracted article, indicate the article's retracted status in the References list and also include a citation and full reference for the retraction notice.

Response: We reviewed the reference list. We corrected and completed some references. We also removed the retracted references and replaced them with the relevant current references, so the revised manuscript does not include retracted articles. We also added new references as we edited the content following the Reviewer's comments.

<u>Responses to Reviewer's comments to the Author</u>

Reviewer #1: Thank you for the opportunity to review this interesting paper. The paper presents two studies investigating disadvantaged families in the Republic of Ireland. To provide context for my comments I will make suggestions under headings.
Introduction
You note that restrictions on public life can have a negative impact on individual and family lives. Can you specify some of these impacts because you then go on to note research about the start of the pandemic but I was unsure if the data presented in that section relates to the mandated closures (lockdown) or the pandemic more broadly. (looking for more specificity in your statements through this section).

Response: We added a paragraph (which corresponds to the second one of the introduction) on the impacts of the pandemic more broadly. In the following paragraph, we sought to provide more details on the impacts of the Covid-19 containment and mitigation measures found in the referenced studies.

Reviewer #1: I’m curious about the decision to add both studies to your submission. The studies relate in topic but are not clearly consecutive or mixed methods where one informs the other. Either they have but it is not clear or you need to justify the decision in your paper.

Response: At the end of the Introduction, we added a justification for adding both studies to the same manuscript. While the studies did not inform each other, we considered that both could provide a comprehensive picture of the experiences of families living in areas with a similar deprivation profile and inform policies and practices affecting these families.

Reviewer #1: Study 1
Uneven gender distribution that should be reflected in your discussion and comments.

Response: In the Discussion of the results of Study 1, we added that the study sample had an uneven gender distribution, being mainly constituted by women. We also added that only women reported a Negative impact on parents' emotional and psychological well-being, and that it was not possible to analyse if gender was a predictor of this experience.

Reviewer #1: I would consider adding reflexivity statements to both of your studies rather than just a statement regarding experience in the methodology.

Response: We removed the information on the authors from the Data analysis section and included it in a new section entitled Reflexivity, which we created within the Methodology of each study. In the Reflexivity section, we included information on which author/s conducted the data collection and respective credentials, occupation at the time of the study, gender, experience and training. We also added a reflection on personal and contextual aspects shaping research.

Reviewer #1: line 306 you allude to positive change seen in other research but don’t specify or consider how/why? How does your specific group compare?

Response: In the Discussion of the results of Study 1, we added information on who the participants were and which positive changes were reported in the referenced studies.

Content that was originally in the General discussion (addressing both studies) was moved to the Discussion of results of Study 1 as a way of avoiding repeating content.

Reviewer #1: The discussion largely repeated the results with brief comments made regarding the implications. You missed an opportunity to explore these alignment with research and recommendations specific to each study (though I note you include a larger discussion later). My advice is reduce replication of results section and extend implications and considerations in study 1 discussion.

Response: In the Discussion of results of Study 1, we sought to reduce replication of results and extend considerations on previous research and potential implications for policy and practice aiming to support families.

Reviewer #1: Study 2
This is a difficult group to sample and you are commended for your efforts. Clarity earlier in the paper about the differentiation of focus groups by age might be useful as it is covered in the discussion but definitely made my list of questions early.

Response: In the Participants section of Study 2, we added information on the ages of the children who participated in the Focus Group Discussions.

Reviewer #1: Results
All there groups appear to be presented together. This needs to be discussed and if sub themes are not addressed by all groups this identified. For example, it seems aspects of educational challenges due to device access were not addressed by children (or you don't have examples of them). Given you have decided to reflect all stakeholders together I suggest you qualify the groups that spoke to each theme and sub theme so we can better appreciate the views of all stakeholders as unique groups not just as a collective.

Response: In the Results section of Study 2, we added information about the themes, sub-themes and groups of participants contributing to each theme and sub-theme. Within themes and sub-themes, we have grouped quotes by participant group, that is children's quotes together, parents' quotes together and service providers' quotes together.

Reviewer #1: Some of the quotes are overly long and could be synthesised but still be

representative. Also in some sections the quotes do not directly address the comments above it.

Response: In the Results section of Study 2, we have shortened the quotes where possible, by removing some words not necessary. However, we were also conscious of trying to ensure the same meaning as conveyed by participants. We have removed quotes we felt did not address the comments above them. We have tried to replace them with quotes relevant to the points. We also added some quotes to better illustrate our findings.

Reviewer #1: 648 I suggest you draw specific attention to technology and WIFI access as clear examples of the divide you speak of

Response: In the General discussion, we added that families in disadvantaged contexts might be less likely to have adequate broadband and hardware, and extended considerations on the potential implications for supporting families.

Reviewer #1: 653 information as you suggest may not be enough without concrete resourcing. I would also note that international studies suggest that thee vulnerable groups are less likely to return to school so some of the 'impact' data you speak of is already there to reference.

Response: In the General discussion, we sought to highlight the importance of concrete resources (such as digital tools) to support families experiencing pandemic-related uncertainty, and measures to support vulnerable groups who are less likely to return to school.

Reviewer #1: Overall an interesting paper that really just needs elaboration, specificity and qualification in some areas.
A pleasure to read.

Response: We thank the Reviewer for the constructive and clear feedback. We consider that the comments helped us improve the manuscript's elaboration, specificity and qualification.
